# DLL3 Is a Prognostic and Potentially Predictive Biomarker for Immunotherapy Linked to PD/PD-L Axis and NOTCH1 in Pancreatic Cancer

**DOI:** 10.3390/biomedicines11102812

**Published:** 2023-10-17

**Authors:** Carlos Lacalle-Gonzalez, Maria Florez-Cespedes, Lara Sanz-Criado, Michael Ochieng’ Otieno, Edurne Ramos-Muñoz, Maria Jesus Fernandez-Aceñero, Luis Ortega-Medina, Jesus Garcia-Foncillas, Javier Martinez-Useros

**Affiliations:** 1 Department of Medical Oncology, Fundación Jiménez Díaz University Hospital, 28040 Madrid, Spain; carlos.lacalle@quironsalud.es; 2 Faculty of Engineering, Imperial College, London SW7 2AZ, UK; maria.florez-cespedes17@imperial.ac.uk; 3 Translational Oncology Division, Oncohealth Institute, Fundacion Jiménez Díaz University Hospital, 28040 Madrid, Spain; lara.sanzcriado@quironsalud.es (L.S.-C.); mikeotson@yahoo.com (M.O.O.); 4 Biomarkers and Therapeutic Targets Group and Core Facility, RICORS2040, EATRIS, Ramón y Cajal Health Research Institute, (IRYCIS), C/Carretera Colmenar Km 9,100, 28034 Madrid, Spain; edurneramosmunoz@gmail.com; 5 Pathology Department, Clinico San Carlos University Hospital, C/Profesor Martin Lagos, 28040 Madrid, Spain; mgg10167@gmail.com (M.J.F.-A.); luis.ortega@salud.madrid.org (L.O.-M.); 6 Area of Physiology, Department of Basic Health Sciences, Faculty of Health Sciences, Rey Juan Carlos University, 28922 Madrid, Spain

**Keywords:** pancreatic ductal adenocarcinoma, delta-like protein 3, NOTCH receptors, prognostic, survival, immunotherapy

## Abstract

Pancreatic ductal adenocarcinoma (PDAC) is an aggressive neoplasm with very poor patient survival outcomes despite available treatments. There is an urgent need for new potential treatment options and novel biomarkers for these patients. Delta-like canonical Notch ligand 3 (DLL3) interacts with the Notch receptor and causes inhibition of Notch signaling, which confers a survival advantage to PDAC cells. Thus, DLL3 expression could affect cell survival, and its inhibition could increase a patient’s survival. To test this hypothesis, a survival analysis was conducted using the progression-free and overall survival from two independent datasets of PDAC patients, with one using mRNA z-score levels and the other using the Hscore protein expression level; both were carried out using a log-rank test and plotted using Kaplan–Meier curves. DLL3 at the mRNA expression level showed an association between high mRNA expression and both a longer progression-free survival (PFS) and overall survival (OS) of patients. Then, we designed a retrospective study with resected PDAC samples. Our primary objective with this dataset was to assess the relationship between PFS and OS and DLL3 protein expression. The secondary assessment was to provide a rationale for the use of anti-DLL3-based treatments in combination with immunotherapy that is supported by the link between DLL3 and other factors that are involved in immune checkpoints. The survival analyses revealed a protective effect of high DLL3 protein expression levels in both PFS and OS. Interestingly, high DLL3 protein expression levels were significantly correlated with PD-L1/2 and negatively correlated with NOTCH1. Therefore, DLL3 could be considered a biomarker for better prognosis in resectable PDAC patients as well as a therapeutic biomarker for immunotherapy response. These facts set a rationale for testing anti-DLL3-based treatments either alone or combined with immunotherapy or other NOTCH1 inhibitors.

## 1. Introduction

Pancreatic ductal adenocarcinoma (PDAC) arises from the ductal epithelium of the gland and accounts for nearly 80% of all pancreatic cancers. Early identifiable symptoms of pancreatic cancer such as painless jaundice, weight loss, or abdominal pain that spreads to the sides or back are rare [1]. This results in a late-stage diagnosis, making pancreatic cancer one of the most lethal forms of cancer [1]. New diagnostic efforts are focused on next-generation sequencing reaching a 95% sensitivity and 100% specificity for cystic precursors and an 82% sensitivity and 100% specificity for advanced neoplasia [2]. The incidence of PDAC as measured by age-standardized rates is highest in Europe (7.7 per 100,000 inhabitants) and North America (7.6 per 100,000 inhabitants). It is more prevalent in men (5.5 per 100,000 inhabitants; 243,033 total cases) than in women (4.0 per 100,000 inhabitants; 215,885 total cases). One of the causes of this sex difference is the higher exposure to main risk factors such as tobacco and alcohol in the male population [3]. The incidence also increases with age due to prolonged exposure to risk factors (such as inflammation, accumulation of mutations, and a declined DNA repair mechanism), making the disease most prevalent in people over 70 years old [4,5].

According to GLOBOCAN 2020, PDAC accounts for as many deaths (466,000) as cases (496,000) and is the seventh leading cause of cancer death in both sexes worldwide [6]. In the United States, data from the National Cancer Institute reveals that the 5-year survival rate for pancreatic cancer is 32% for individuals diagnosed at the local stage, 12% for stage III cases, and only 3% for those diagnosed at stage IV. A majority of cases (52%) are diagnosed at the advanced stage [7]. By 2040, it is projected that 355,317 new cases will occur. Moreover, in a study involving 38 European countries, it was projected that pancreatic cancer will surpass breast cancer as the third leading cause of cancer death by 2025 [8].

Currently, the best treatment option is whole tumor resection. However, due to the late diagnosis nature of pancreatic cancer, only 15 to 20% of patients are eligible for this procedure. Moreover, even after a complete resection, patients often still have a poor prognosis [9]. Multiple strategies have been explored to improve outcomes for resected PDAC patients, with one of the most effective solutions being adjuvant chemotherapy [10]. The first trial to demonstrate the benefit of adjuvant chemotherapy was CONKO-1 [11], in which randomized patients received six cycles of gemcitabine at 1250 mg/m^2^ on days 1, 8, and 15 every 4 weeks *versus* observation. The primary endpoint showed an improvement in the median progression-free survival (PFS, 13.4 months) in the gemcitabine group (95% confidence interval (CI): 11.4–15.3), compared with 6.9 months in the control group (95%CI: 6.1–7.8; *p* < 0.001). Unfortunately, this trial failed to demonstrate an increase in overall survival (OS). After the CONKO-1 trial, several trials tested different regimens of chemotherapy to improve outcomes for these patients. Two recent trials (ESPAC-4 and PRODIGE-24), which evaluated the role of adjuvant chemotherapy in PDAC, have led to a change in the standard of care [12,13]. The ESPAC-4 trial [9] included adult patients who had undergone complete surgery and were later randomized to gemcitabine alone or gemcitabine plus capecitabine for 6 months. This trial confirmed a 2,5 month increment in overall survival in resected PDAC patients. The PRODIGE-24 trial [10] evaluated the role of a modified regimen of 5-fluorouracil/leucovorin/irinotecan/oxaliplatin (mFOLFIRINOX). This regimen surpassed 6 months survival after resection compared with gemcitabine alone. 

Currently, the standard of care for patients after the resection of their PDAC tumors is combined chemotherapy (mFOLFIRINOX or gemcitabine/capecitabine). Without a direct comparison between these two regimens, it seems that mFOLFIRINOX could be the best treatment. Another treatment modality that is incorporated in resectable or locally advanced pancreatic cancer is radiation therapy. Although it was unable to demonstrate overall survival benefit alone or in combination with chemotherapy [14], it could be useful to sterilize vessel margins, enhance the likelihood of a margin-negative resection, and/or provide adequate local control to prevent or delay progression and local disease recurrence [15].

In the metastatic setting, another combination composed by gemcitabine and nano albumin-bound paclitaxel (Nab-paclitaxel) increased both progression-free survival (PFS) and overall survival (OS) [16]. As there was no available direct comparison between gemcitabine/Nab-paclitaxel and mFOLFIRINOX until 2023, an indirect comparison suggested a slightly greater efficacy and higher toxicity with mFOLFIRINOX. Therefore, current guidelines recommend mFOLFIRINOX or gemcitabine/Nab-paclitaxel as first-line treatment in metastatic patients with ECOG 0–1 and in patients younger than 75 years old. Gemcitabine alone is reserved for ECOG 2 patients or patients older than 75 years old [17,18].

In January 2023, the results of the NAPOLI-3 trial [19], a randomized, open-label phase 3 study, were published; the study compared liposomal irinotecan/5-fluorouracil/leucovorin/oxaliplatin (NALIRIFOX) *versus* gemcitabine/Nab-paclitaxel in treatment-naïve patients with metastatic PDAC. Median OS was 11.1 months in the NALIFIROX arm compared with 9.2 months in the gemcitabine/Nab-paclitaxel arm (*p* = 0.04). PFS was also significantly improved (7.4 months *vs.* 5.6 months; *p* = 0.0001). Considering these results, NALIRIFOX will likely be the recommended first-line chemotherapy in future guidelines. 

Additionally, neoadjuvant treatment can be an option in selected patients prior to surgery and after an initial tumor reduction. The SWOG S1505 [20] phase II trial with 102 patients compared mFOLFIRINOX *versus* gemcitabine/Nab-paclitaxel in this perioperative setting. The trial failed to demonstrate an improved OS in perioperative chemotherapy (prespecified endpoint of a 58% two-year survival resulted in a 47% and 48% two-year OS in the mFOLFIRINOX and gemcitabine/Nab-paclitaxel group, respectively). Noteworthy, resectability rates were high with both regimens (73 *versus* 70%), although more patients who received gemcitabine/Nab-paclitaxel had a complete or major pathologic response (42 *versus* 25%).

Despite all these chemotherapy treatment efforts, survival outcomes for PDAC patients remain poor. New treatment strategies like immunotherapy (mainly PD-1/PD-L1 antibodies) have completely changed the treatment paradigm and prognosis in other aggressive tumors like lung cancer [21], bladder cancer [22], or triple-negative breast cancer [23], even eliminating the need for chemotherapy. However, immunotherapy did not show significant clinical efficacy outside of the mismatch repair deficiency setting in most phase I and II clinical trials in PDAC [24,25]. Therefore, PDAC urgently needs new treatment strategies to improve current chemotherapy benefits, such as targeting specific signaling pathways. Precisely, the Notch signaling pathway has a unique importance in PDAC due to its connection with other pathways that are associated with pancreatic embryonic development and differentiation [26].

The Notch receptor family (NOTCH1–4) consists of a transmembrane cell surface receptor that transduces the juxtacrine signals of the transmembrane ligands delta-like canonical Notch ligand (DLL1/3/4) and jagged canonical Notch ligand (JAG1/2). DLL/JAG agonistic ligands trigger sequential proteolytic cleavage of Notch receptors through ADAM10/17 and gamma-secretase, which generates three parts: the Notch extracellular domain, transmembrane domain, and intracellular domain. The last part is translocated to the nucleus and promotes the expression of several survival genes like *c-MYC*, *NANOG*, *SOX2*, and *OCT-4* that are relevant for pancreatic cancer stem cell renewal [27,28,29,30].

DLL3, one of the ligands of the Notch signaling pathway located in the cell membrane of tumor cells, is often expressed in neuroendocrine tumors and exerts an inhibition of DLL1/NOTCH interaction, causing an inhibition of the Notch pathway [30,31]. Under physiological conditions, DLL3 is mostly found in the Golgi apparatus; however, if overexpressed, it can reach the cell membrane and could be useful for Notch pathway inhibition [32,33]. In other tumors like small-cell lung cancer, DLL3 is upregulated and maintains growth and neuroendocrine differentiation [34]. Furthermore, DLL3 expression is more abundant in neoplastic cells, especially in small-cell lung cancer and other neuroendocrine neoplasms, as opposed to healthy cells where they do not exert any expression except for in the testis and central nervous system. Despite these results, the role of DLL3 in other non-neuroendocrine tumors like PDAC remains unexplored. 

Overall, several efforts have been made to develop new agents against DLL3, e.g., antibody–drug conjugates (Rovalpituzumab-tesirine, Abbvie), bispecific/DLL3-targeting T-cell engagers (AMG 757, Amgen/ HPN328, Harpoon Therapeutics), and the first chimeric antigen receptor T (CAR-T) cellular therapy (AMG 119, Amgen). Rovalpituzumab tesirine is a novel antibody–drug conjugate that targets DLL3 protein and delivers tesirine (pyrrolobenzodiazepine dimer toxin very similar to anthracycline) inside the cell. After internalization, tesirine is released and causes DNA damage in tumor cells that overexpress DLL3 [35]. In small-cell lung cancer, the drug demonstrated promising results in an initial phase I/II trial [36]. Phase III trials focused the activity on DLL3-high populations (≥75% expression on immunohistochemistry); unfortunately, it failed to demonstrate a survival benefit against second-line standard chemotherapy in small-cell lung cancer with an adverse safety profile [37,38]. This toxicity could be related to the premature cleavage of the linker causing a systemic release of tesirine. HPN328 results from an ongoing phase I clinical trial showed one confirmed partial response, and 33% of small-cell lung cancer patients had a 30% reduction in target lesions across all doses [39]. AMG 757, a bispecific T-cell engager molecule, binds both DLL3 and CD3 cells leading to T-cell-mediated cytotoxicity. This drug demonstrated a promising activity with a good safety profile in a phase I clinical trial with 107 patients enrolled, showing an objective response rate of 23.4%, 23/107 partial responses, and 2/107 complete responses [40]. 

Due to the lack of effective treatment options in PDAC, there is an urgent need to explore new biomarkers, especially if they have a therapeutic association. As DLL3’s prognostic and therapeutic implications are not formally explored in PDAC, in this article we provide a survival analysis and insight into the potential use of DLL3 as a predictive biomarker for immunotherapy as well as other anti-DLL3-based strategies.

## 2. Materials and Methods

### 2.1. TCGA Dataset Analysis

The TCGA Firehose Legacy database, composed of 186 tumor samples (accessed on 18 June 2022), was used to determine whether DLL3 mRNA expression could have an association with survival. Values of expression from the TCGA dataset were taken using cBioPortal [41,42]. Then, expression data was converted to a z-score following the formula z-cores = (expression in each tumor sample—mean expression of all tumor samples)/standard deviation of expression of all tumor samples). To assess the association between high and low DLL3 expression and overall and progression-free survival, we determined a cut-off point with a receiver operating characteristics curve. However, as the receiver operating characteristics curve did not show a clear cut-off point, the third quartile (percentile 75) of the z-score was used as the best cut-off point to divide patients into low and high DLL3 expression. 

### 2.2. Patient Samples

All patients who underwent duodenopancreatectomy from 2007 to 2013 at the Hepatobiliary and Pancreatic Surgery Unit (General and Digestive Tract Surgery Department, Clínico San Carlos Hospital) were assessed for eligibility, and samples were provided by its institutional Biobank (B.0000725; PT17/0015/0040; ISCIII-FEDER). The institutional review board (IRB) of University Hospital Clinico San Carlos evaluated the present study, granting approval on 10 March 2017 with approval number nº 17/091-E. The main criteria for eligibility were patients with a final histopathological diagnosis of localized pancreatic ductal adenocarcinoma who underwent surgery. Tumors were surgically resected and were formalin-fixed and paraffin-embedded immediately for pathologic diagnosis. To assess the survival analysis, only patients with a confirmed pathological diagnosis of PDAC and available data for PFS and OS were included in the study. 

### 2.3. Tissue Microarray

Tissue microarrays were constructed using available formalin-fixed and paraffin-embedded tumor samples. Tissue microarrays contained 2 cores per patient using the MTA-1 tissue arrayer (Beecher Instruments, Sun Prairie, WI, USA). A hollow needle was used to obtain a tissue core of 1 mm in diameter from selected tumor regions in formalin-fixed and paraffin-embedded tissues. These tissue cores were then inserted in a recipient paraffin block with precise spacing, resembling an array pattern. Sections from this paraffin block were cut in a microtome and mounted on a microscope slide to be analyzed using immunohistochemistry. 

### 2.4. Immunohistochemistry 

Staining was conducted in 2 μm sections. Firstly, the slides were deparaffinized by incubation at 62 °C for 10 min and incubated for antigen retrieval using the PT-Link (Dako, Glostrup, Denmark) for 20 min at 97 °C in a high-pH buffered solution (EnVision Dako kit). The samples were then incubated with peroxide (EnVision Flex peroxidase blocking reagent) to block the endogenous peroxidase. The biopsies were stained overnight with diluted antibodies against DLL3 (1:100, ab229902, Abcam, Cambridge, UK), PD-L1 (1:1, PD-L1 IHC 22C3 pharmDx assay (Agilent Technologies, Carpinteria, CA, USA)), PD-L2 (1:100, ref: ab214221, Abcam, Cambridge, UK), and NOTCH1 (1:100, D1E11, ref: 36085, Cell Signaling, Danvers, MA, USA) followed by incubation with the appropriate anti-Ig conjugated with horseradish peroxidase (Anti-mouse/Anti-rabbit EnVision FLEX-HRP Labeled Polymer; Dako, Glostrup, Denmark) for 20 min. Visualization of the sections was carried out with 3,3′-diaminobenzidine (DAB, Dako, Glostrup, Denmark) as a chromogen for 5 min. Hematoxylin (Harris’ Hematoxylin, Sigma-Aldrich, San Luis, MO, USA) was used for counterstaining for 2 min. All antibodies and anti-Ig horseradish peroxidase-conjugated antibodies presented high specificity, and no positiveness resulted from these antibodies individually. To determine the best immunohistochemistry conditions and maximize the specificity of antibodies, different healthy control tissues were used as positive controls according to “The Human Protein Atlas” (http://www.proteinatlas.org (accessed on 12 October 2023)). The evaluation of stainings has been performed by two independent pathologists (M.J.F.-A. and L.O.-M.).

### 2.5. Quantification of Immunohistochemistry

The evaluation of the immunoreactivity of the different antibodies was carried out using a semiquantitative method called HistoScore, which considers the intensity of staining and the percentage of positively stained cells. The HistoScore (Hscore) ranged 0–300 and multiplied the percentage of positively stained cells for each of the low, medium, or high staining intensities. The final score was determined using the following formula.

$$HScore={(1\times \%}_{Low})+\left(2\times {\%}_{Medium}\right)+\left(3\times {\%}_{High}\right)$$

Tumor stroma is actively related to PDAC, and it plays a crucial role in the chemoresistance and aggressiveness of this tumor; therefore, DLL3 expression was also determined and analyzed in the tumor stroma. 

For PD-L1 cells, the combined positive score (CPS) was calculated according to the following formula similarly to how it is performed in the clinical setting, with a specific threshold of ≥1%:

$$CPS=\frac{\left(Number\;positive\;PD-L1\;cells\right)}{\left(Total\;number\;of\;tumor\;cells\right)}\times 100$$

### 2.6. Statistical Analysis

Progression-free survival was defined as the interval between the dates of surgery and recurrence (local or distant). Overall survival was defined as the interval between the dates of surgery and death. In order to select a clear cut-off point to separate low from high DLL3 expression in both the tumor and stroma, we performed a receiver operating characteristics curve analysis; however, as it did not show a clear cut-off point, we performed a Kolmogorov–Smirnov test. The test indicated a normal distribution of tumor DLL3, so we decided to use the median as the cut-off point in both cases. Survival according to DLL3 was shown using Kaplan–Meier plots and analyzed using the log-rank test. The univariate Cox proportional hazards model was used to assess the hazard ratios and confidence intervals of DLL3 and other clinicopathological variables. Only variables that are considered statistically significant in the univariate analysis were included in the multivariate analysis. Descriptive and Kolmogorov–Smirnov normality tests were used to study the normal distribution of the variables in the patient cohort. The correlation between PD-L1, PD-L2, POLE, NOTCH1, and DLL3 in tumor or stroma was performed using the Pearson test for parametric variables or using the Spearman test for non-parametric ones and was interpreted according to Cohen *et al.* 1988 [43]. Statistical associations were explored using crosstabs and analyzed using a χ2 test or, in the event of frequency values < 5, using a Fisher exact test. IBM-SPSS statistics v.20 was used to calculate the primary, secondary, and exploratory endpoints. Statistically significant results were considered if *p*-value < 0.05. 

## 3. Results

### 3.1. High DLL3 mRNA Expression Is Associated with Better Prognosis in PDAC Patients

A total of 186 patients were included; a total of 103 patients were male (55%) and 83 were female (45%). The origin site of the primary tumor was the pancreas in all patients, and 177 (95.2%) had a PDAC. The median age at diagnosis was 65 years (range from 35 to 88 years), and 89 patients (48%) were diagnosed above 65 years old. The eighth edition of the TNM tumor staging system was assessed. There were 20 stage I (10.8%) patients, 148 stage II (79.5%) patients, 4 stage III (2.2%) patients, and 6 stage IV (3.2%) patients; 8 (4.3%) patients were lost. The survival analysis was assessed using patients with available data for DLL3 expression and OS (n = 175) or PFS (n = 139).

**Figure 1:**
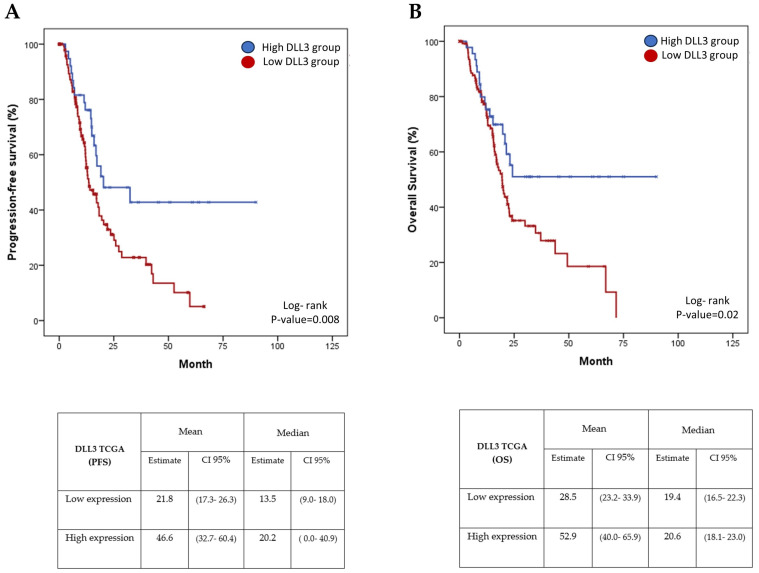
Survival analyses according to DLL3 mRNA expression. (**A**) Progression-free survival analysis according to DLL3 mRNA expression. (**B**) Overall survival analysis according to DLL3 mRNA expression. The blue lines denote the high DLL3 expression arm. The red lines correspond to the low DLL3 expression patients.

Overall, 134 patients were classified as having a low DLL3 expression of mRNA and 45 were classified with a high DLL3 expression of mRNA. From the 139 patients evaluated for PFS, those with a lower expression of DLL3 showed a mean PFS of 21.8 months (95%CI: 17.3–26.3 months) and median PFS of 13.5 months (95%CI: 9.0 to 18.0 months) compared with the mean PFS of patients with high DLL3 expression (46.6 months; 95%CI: 32.7–60.4 months) and the median PFS for DLL3 high expression, which was 20.2 months (95%CI: 0–40.9 months). Interestingly, the median PFS differences from high to low DLL3 expression samples was 6.7 months (Figure 1A, *p*-value = 0.008).

Furthermore, the mean OS in the high expression DLL3 group was 52.9 months (95%CI: 40.0–65.9 months), and the median OS in those cases was 20.6 months (95%CI: 18.1–23.0 months); meanwhile, the mean OS of the low expression DLL3 group was 28.55 (95%CI: 23.2–33.9 months), the median OS of the low expression DLL3 group was 19.4 (95%CI: 16.5–22.3 months). Despite no significant differences being seen in the median overall survival results, there was an astonishing mean OS difference of 24.4 months observed (*p*-value = 0.02) (Figure 1B).

The Cox hazard model for PFS and OS also demonstrates a lower risk in DLL3 high expression. As expected, other co-variates appeared to be statistically significantly associated with poor survival, such as positive margins of resection (*p* = 0.004 for both PFS and OS) and high tumor grade (*p* = 0.03 for OS) (Table 1). Therefore, high DLL3 expression at the mRNA level could be considered a good prognostic biomarker for PDAC and could be easily quantified using real-time PCR with the resected tumor in clinical practice.

### 3.2. Population Characteristics of the Validation Set

**Figure 2:**
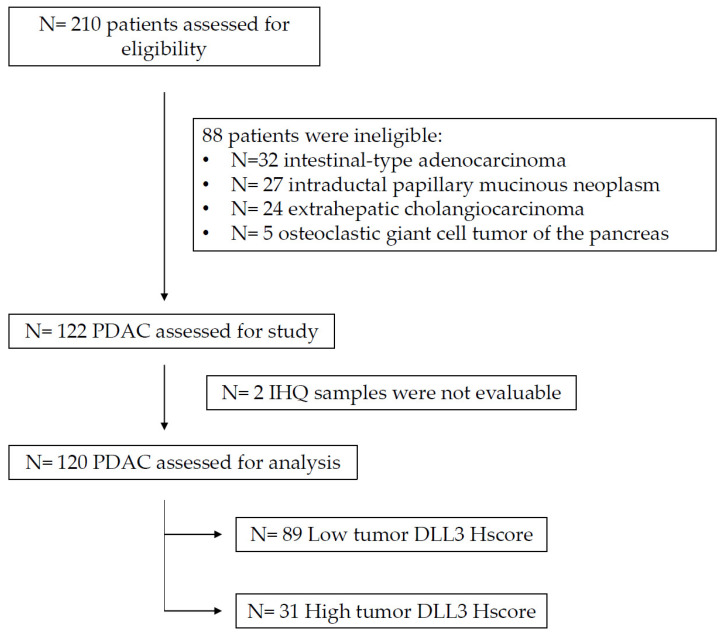
Flowchart with initial sample size and all exclusion criteria to obtain a homogeneous population of PDAC to proceed with the analyses.

From 2007 to 2013, a total of 210 patients underwent duodenopancreatectomy. From the pathologic report, 32 were intestinal-type adenocarcinoma, 27 were intraductal papillary mucinous neoplasm (16 with associated invasive carcinoma), 24 were extrahepatic cholangiocarcinoma, and 5 were classified as osteoclastic giant cell tumor of the pancreas. Overall, 122 of the initial 210 patients were diagnosed as PDAC and were therefore admitted to analysis. Unfortunately, two samples were not evaluable clinicopathologically; thus, the sample size was composed of 120 patient samples (Figure 2). 

Of these samples, 68 (55.7%) were female and 54 (44.3%) were male. The median age at the time of diagnosis was 72 years old (ranging from 44 to 86 years), and 89 (73%) of the patient were older than 65 years old at diagnosis. Adjuvant treatment was received by 50 (41%) of the patients. Inside the histologic characteristics analyzed, 41 (33.6%) had low-grade PDAC while 77 (63.1%) had high-grade PDAC; vascular and perineural invasion were present in 53 (43.4%) and 91 (74.6%) patients, respectively. Tumor margin status after the surgery was R0 (complete resection) in 65 (53.3%), R1 (microscopic residual tumor) in 41 (33.6%), and R2 (macroscopic residual tumor) in 0 (0%) patients. Using the eighth edition of the TNM tumor staging (published in 2016), most of the patients were stage II/IIA (15 (12.3%)) and IIB (41 (41.8%)), followed by stage I/IA (18 (14.8%)) and IB (13 (10.7%)); finally, 18 (14.8%) patients were stage III. All clinicopathological characteristics of this homogeneous cohort are summarized in Table 2.

### 3.3. DLL3 Protein Expression Is a Prognostic Biomarker after Resection in PDAC

**Figure 3:**
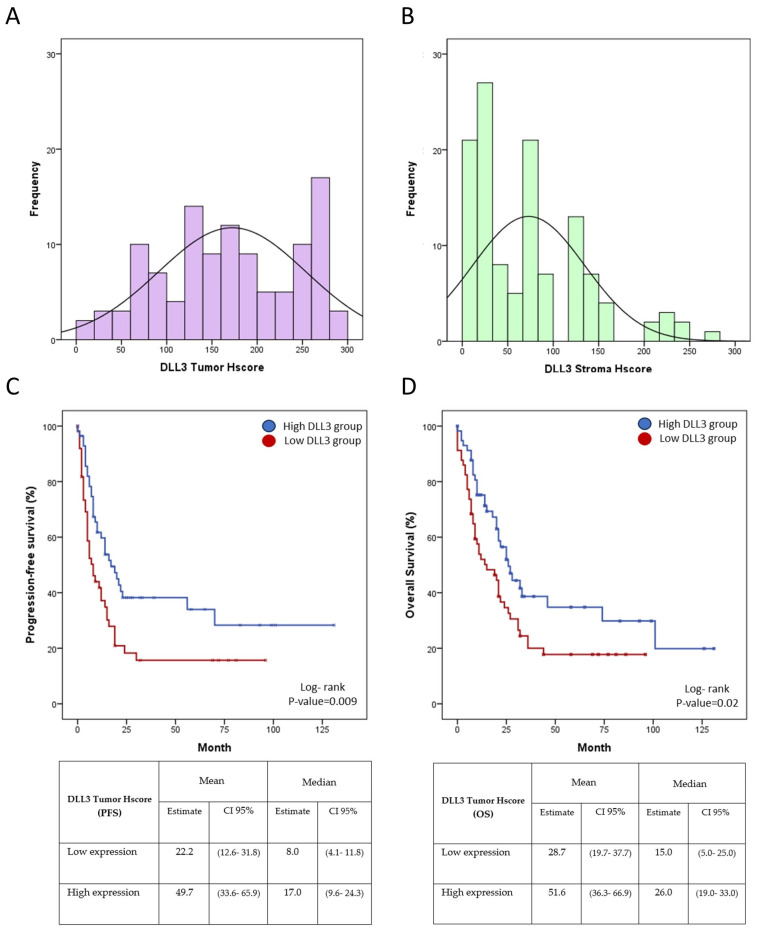
Survival analyses according to DLL3 protein expression in tumor tissues. Histograms of DLL3 expression in the tumor (**A**) and in the stroma (**B**). (**C**) Progression-free survival analysis according to DLL3 protein expression. (**D**) Overall survival analysis according to DLL3 protein expression. The blue lines denote the high DLL3 expression arm. The red lines correspond to the low DLL3 expression patients.

After DLL3 mRNA expression demonstrated a good correlation with better patient outcomes, we proceeded to evaluate survival according to the DLL3 protein in a validation cohort. Here, the percentage of DLL3 expression in tumor cells ranged from 10 to 100%, with a mean and median value of 64,6% and 70%, respectively. The DLL3 intensity of expression was low in 6 (5%), medium in 35 (29.2%), and high in 79 (65.8%) samples from a total of 120 samples. Kolmogorov–Smirnov test supported a normal distribution of DLL3 expression in the tumor samples (*p*-value = 0.113) (Figure 3A).

Interestingly, the DLL3 immunohistochemical expression of the stroma was higher than expected in normal pancreatic tissues. The expression shows a percentage of expression that ranges from 5 to 90%, with a mean and median value of 35.7% and 35%, respectively. DLL3 intensity was low in 41 (33.8%), medium in 56 (46.2%), and high in 24 (20%) samples from a total of 120 samples. The Kolmogorov–Smirnov test does not support a normal distribution of DLL3 expression in the stroma samples (*p*-value < 0.001) (Figure 3B). Low DLL3 tumor expression showed a shorter median PFS of 8 months (95%CI: 4.1–11.8 months) compared with the median PFS of the high DLL3 expression arm, which was 17 months (95%CI: 9.6–24.3 months) (*p*-value = 0.009). Also, the mean PFS of the low DLL3 expression group was 22.2 months (95%CI: 12.6–31.8 months), while the mean PFS of the high DLL3 expression group was 49.7 months (95%CI: 33.6–65.9 months) (Figure 3C). The median OS in the high expression group was 26 months (95%CI: 19–33 months) compared with the low expression group, which exhibited 15 months (95%CI: 5–25 months). Moreover, the mean OS of the low DLL3 expression group was 28.7 months (95%CI: 19.7–37.7 months), while the mean OS of the high DLL3 expression group was 51.6 months (95%CI: 36.3–66.9 months) (*p* = 0.02) (Figure 3D). 

Subsequently, to compare the potential prognosis value of DLL3 tumor expression with the other registered clinical variables, a Cox proportional hazards model was performed. The univariate analysis for PFS confirmed that patients with high DLL3 tumor expression showed a lower risk of recurrence after surgery compared with those with low DLL3 (HR: 0.55; 95%CI: 0.34–0.87; *p* = 0.011). Furthermore, the Cox univariate hazard model for PFS showed that symptoms, size (≤2 cm *vs.* >2 cm), stage (I *vs.* II), and lymph nodes involvement were statistically significantly associated with PFS; however, in the multivariate Cox analysis, only tumor DLL3 expression remained statistically significant (HR: 0.41; 95%CI: 0.23–0.71; *p* = 0.02) (Table 3). 

Additionally, the Cox univariate proportional hazard model for OS also demonstrated that high DLL3 tumor expression is related to a lower risk of death compared with those having low DLL3 expression in tumor cells (HR 0.59; 95%CI: 0.37–0.93; *p* = 0.024) (Table 3). Other variables in the OS Cox univariate hazard model like symptoms at diagnosis, size (≤2 cm *vs.* >2 cm), and stage (I *vs.* II) showed a statistically significant association with a shorter OS. Interestingly, upon multivariate analysis of OS, only DLL3 expression in tumor appeared to be statistically significant (*p* = 0.004) (Table 3). Taken together, DLL3 expression at the protein level could be considered a good prognostic biomarker after resection in PDAC patients.

We also evaluated the expression of DLL3 in the stroma of the patient samples. DLL3 expression in the stroma revealed no statistically significant association with PFS (*p* = 0.358; Supplementary Figure S1A) or with OS (*p* = 0.718; Supplementary Figure S1B). Therefore, only the expression of DLL3 in the tumor cells at the mRNA or protein level could be considered as a prognostic biomarker for PDAC.

To verify whether DLL3 expression in the tumor or stroma could be associated with any of our registered clinicopathological variables, a chi-square test was performed. In the crosstabs, there were statistically significant associations between DLL3 expression in the tumor and perineural invasion (*p* = 0.007) and lymph node involvement (*p* = 0.029) (Table 4). As expected, an association between DLL3 expression in tumor and in stroma was confirmed (*p* = 0.018) (Table 4).

Furthermore, a statistically significant low correlation was found between DLL3 tumor expression and the number of lymph nodes affected (ρ = 0.18; *p* = 0.049). In contrast, DLL3 protein expression in stroma was moderately correlated with tumor size (ρ = 0.276; *p* = 0.005). As expected, DLL3 tumor expression was moderately significantly correlated with DLL3 expression in stroma (ρ = 0.4; *p* = 0.0001). 

### 3.4. DLL3 Tumor Expression Is a Potential Biomarker for Immunotherapy Response

DLL3’s relationship to immunotherapy biomarkers is not properly studied in PDAC. For this purpose, PD-L1, PD-L2 and POLE expression was measured in our sample. PD-L1 results in tumor and inflammatory cells were predominantly low, with 0% of expression in 100 (82%) and 104 (85.2%) of all samples and a mean value of 2.4% and 2%, respectively. PD-L2 was slightly more expressed than PD-L1, having a 0% expression in 51 (47.7%) samples and a mean value of 16.3% in the samples. POLE nuclear expression was also low with a median of 10% with a standard deviation of 27.8% and a mean of 24%; it ranges from a minimum of 0% to a maximum of 90%. 

**Figure 4:**
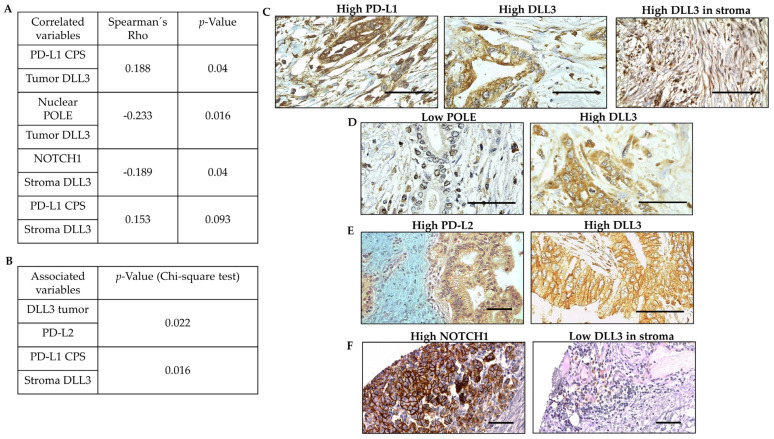
DLL3 is related to other relevant oncogenic therapeutic targets. (**A**) Spearman correlation between DLL3 in tumor or stroma with PD-L1 CPS, POLE, or NOTCH1. (**B**) Statistical association between DLL3 tumor and stroma expression to PD-L2 and PD-L1 CPSs, respectively. (**C**) Representative micrographs from the same patient with high PD-L1 and high DLL3 expression in tumor and in stroma. (**D**) Representative micrographs from the same patient with low POLE and high DLL3 in tumor. (**E**) Representative micrographs from the same patient with high DLL3 in tumor and high PD-L2 expression in tumor. (**F**) Representative micrographs from the same patient with high NOTCH1 and low stroma DLL3. Scale bar is 100 µm.

Here, a low positive correlation was found between DLL3 tumor expression and PD-L1 CPS (%) (ρ = 0.188; *p* = 0.04) (Figure 4A,C) as well as a low negative correlation with POLE nuclear expression (%) (ρ = −0.233; *p* = 0.016) (Figure 4A,D). Thus, tumors that express DLL3 show more PD-L1 expression and less expression of nuclear POLE. 

We also assessed the analysis of DLL3 expression in stroma and found that DLL3 stroma expression exhibited a trend towards significance with PD-L1 (ρ = 0.153; *p* = 0.093) (Figure 4A,C). This result was supported by an association between DLL3 stroma expression and a PD-L1 CPS ≥ 1% (*p* = 0.016) (Figure 4B). Furthermore, an interesting association was found between DLL3 tumor protein expression and PD-L2 expression (*p* = 0.022) (Figure 4B,E). These findings suggest a link between DLL3 expression and the PD/PD-L axis. 

### 3.5. DLL3 Counteracts NOTCH1 Expression in Resected PDAC

NOTCH1 expression in our samples was very low compared with DLL3 expression. A total of 115 (94.3%) samples did not express NOTCH1 with a mean of expression of 2.38% (range: 10–80%). NOTCH1 expression was negatively correlated with DLL3 expression in stroma (ρ = −0.189; *p* = 0.04) (Figure 4A,F). Additionally, a high trend towards significance was also found between DLL3 tumor expression (ρ = −0.154; *p* = 0.09) and NOTCH1 expression. Overall, these negative correlations reinforce the connection between DLL3 in the tumor microenvironment and NOTCH1 (Figure 4F).

## 4. Discussion

The poor prognosis and therapeutic outcomes of resected PDAC are mostly caused by the absence of targeted treatments. PDAC has been ruled out from the benefit of therapeutic revolutions such as immunotherapy. One of the main reasons for the lack of efficacy of immunotherapy in PDAC is the absence of a good predictive biomarker in clinical trials. However, PD-L1 is not the only biomarker for immunotherapy response, and PDAC patients with high microsatellite instability have been shown to benefit from immunotherapy [44]. Delta-like canonical Notch ligand 3 is a novel therapeutic target for neuroendocrine tumors, having a huge field of pharmacologic development that has not been formally studied in non-neuroendocrine neoplasms. The urgent need for new therapeutic targets pushed our team to study DLL3 in a non-neuroendocrine tumor like PDAC.

To the best of our knowledge, this is the first formal study directed at the role of DLL3 in resected PDAC that shows a clear role in overall and progression-free survival. DLL3 seems to have a positive impact on survival, which is probably due to the inhibition of the DLL1–NOTCH cellular interaction [30,31]. The correlation and association of tumoral DLL3 with perineural invasion and lymph node involvement, together with the correlation of stromal DLL3 to tumoral size, lead us to think that the DLL3–NOTCH inhibition mechanism tends to appear late in the history of PDAC and is related to more advanced TNM stages.

As the DLL3 interaction occurs between cells, stroma and tumor cells should be studied together to elucidate all of the interactions between the tumor microenvironment and the tumor cells. Other biomarkers, like PD-L1, started in tumor cells only; however, they were recently reformulated to consider the immune cells in the stroma. Based on this, the CPS was established, and it constitutes the biomarker of choice for guiding immunotherapy in head and neck squamous cell carcinoma [45], gastroesophageal carcinoma [46], triple-negative breast cancer [47], and cervical cancer [48]. One limitation of our study is the lack of metastatic patients to perform a subgroup analysis and explore the role of DLL3 in the population with a worse prognosis. In light of these results, DLL3 should be urgently explored in stage IV patients in future studies. 

**Figure 5:**
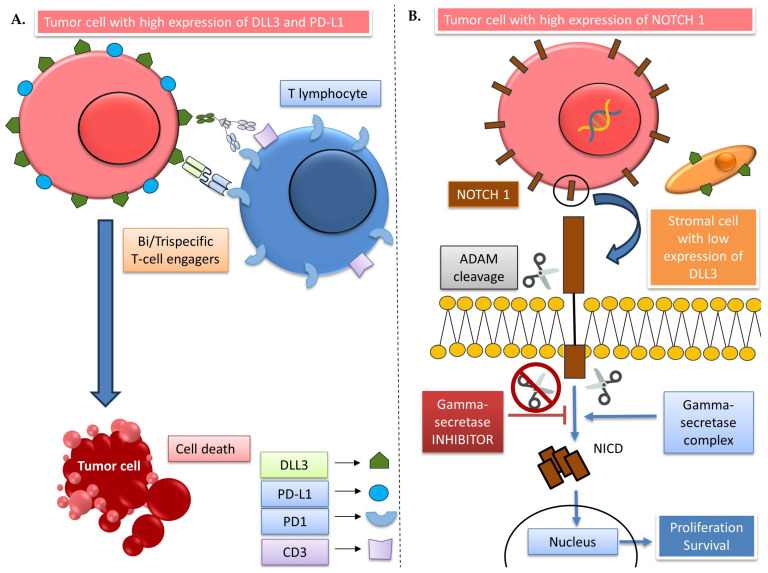
DLL3 status could define different therapeutic approaches in PDAC. (**A**) High DLL3 tumors are associated with more immune infiltrate and high PD-L1 expression, which leads to an inhibition of T lymphocytes. T-cell engagers could break this inhibition and use DLL3 on the surface of tumor cells to trigger a T-cell-mediated death. (**B**) Low DLL3 expression in stroma is related to high NOTCH1 expression that could lead to more proliferation and survival of PDAC cells. Gamma-secretase inhibitors could interfere with NOTCH1 receptor cleavage and avoid the downstream signal through the intracellular domain of the NOTCH protein (NICD).

The regulation of the PD-L1 receptor remains elusive, and to the best of our knowledge, there is no reported association or correlation between DLL3 and PD-L1 expression at the molecular level in PDAC. Only one study in small-cell lung cancer showed a correlation between DLL3 and PD-L1 expression that supports our results [49]. Therefore, the relationship of DLL3 with the PD/PD-L axis is extremely important and could open therapeutic opportunities for PDAC patients. DLL3 expression is related to higher immune cell infiltrates in several tumor types and PDAC [50] (Figure 5A). Indeed, in our sample, a positive correlation between DLL3 expression in tumors and PD-L1 CPS alongwith a positive association with PD-L2 expression guide us to think that high DLL3 PDAC patients have more immune infiltrates with high PD-L1/2 expression. The PD-L1/2 bind to programmed death receptors on T lymphocytes bring an inhibitory signal and lead to an induction of T-cell tolerance to the tumor [51] (Figure 5A). This situation could be reversed with the use of a bispecific T-cell engager that binds to DLL3-PD1 [52,53] or a trispecific T-cell engager that could bind to DLL3-PD1-CD3 (Figure 5A). Some of these drugs are currently in development for small-cell lung cancer; however, they are being neglected for PDAC. In addition, low DLL3 tumors are not likely to express high PD-L1/2 and do not have high immune infiltrates; therefore, the bi/trispecific T-cell engagers could not be a valid therapeutic strategy for this patient subset.

For low DLL3-expressing tumors, a higher Notch signaling pathway activity is expected due to a negative correlation between DLL3 stroma expression and Notch tumor expression. Furthermore, DLL3 stroma and tumor expression are positively correlated with each other. Thus, lower tumor DLL3 expression will be found in the context of low stroma DLL3 expression and, consequently, more Notch activity will be found in those cases. Thus, for the low DLL3 PDAC patients, a higher NOTCH expression is expected, and this higher NOTCH expression could explain the worse prognosis of the low DLL3 expression arm in our cohort; however, it could open a therapeutic opportunity for this poor prognosis subset. A recent review showed that the Notch signaling pathway is important for differentiation of immune cells within the tumor microenvironment. It described how in NK cells, DLL1, DLL4, and Jagged2 together with cytokines stimulate the differentiation of these cells in vitro. DLL1-mediated NOTCH signaling is activated to promote the expression of CD16 and killer Ig-like receptors that are important for cytotoxicity against tumor cell. In macrophages, in vitro models of melanoma show a higher correlation of NOTCH1, NOTCH2, and Hes1 expression in M1 macrophages. In vitro studies point out the importance of NOTCH2 in the differentiation of the innate lymphoid cells [54].

Gamma-secretase inhibitors that act to impede the NOTCH receptor cleavage are also other drugs that have not been fully explored in PDAC. A phase II study of the gamma secretase inhibitor, RO4929097, failed to demonstrate an improvement in the 6-month OS in second-line metastatic patients, although the lack of a good biomarker for properly selecting the patients surely impacted these results [55] (Figure 5B). Other gamma-secretase inhibitors such as MRK-003 show good preclinical activity and also potentiate chemotherapy sensitivity [56,57]. These inhibitors were able to demonstrate clinical benefit in central nervous system malignancies and desmoid tumors [58]. Notwithstanding the foregoing, we propose DLL3 as a biomarker for the therapeutic use of gamma-secretase inhibitors in PDAC patients (Figure 5B).

## 5. Conclusions

This is the first study that demonstrates the positive association between DLL3 expression in the tumor specimen with both progression-free and overall survival in resected pancreatic ductal adenocarcinoma patients. Indeed, exploratory endpoints show that DLL3 tumor expression is positively correlated with PD-L1 CPS, which could contribute to better stratifying patients to receive an anti-PD-L1 treatment. Because we found that NOTCH1 is negatively correlated with DLL3 stroma expression, low DLL3 tumors could be potentially treated with gamma-secretase inhibitors to block NOTCH1. Because anti-PD-L1-based treatments per se have failed to demonstrate a survival benefit in PDAC patients, we propose bi/trispecific drugs that target DLL3, PD-L1, and/or CD3 to improve immunotherapy response in such tumors. Furthermore, the association between DLL3 and lymph node involvement and perineural invasion could address new opportunities for more advanced PDAC, such as in metastatic patients.

## Figures and Tables

**Table 1:** Uni- and multivariate proportional hazard model for both the PFS and OS of patients analyzed according to DLL3 mRNA expression levels.

	Univariate PFS (95%CI)				Univariate OS (95%CI)			
	HR	Lower	Upper	*p*	HR	Lower	Upper	*p*
Age (<65 years *vs.* >65 years)	1.39	0.90	2.16	0.13	1.39	0.93	2.09	0.10
Sex (Male *vs.* Female)	1.17	0.76	1.81	0.45	1.25	0.83	1.87	0.27
Diabetes mellitus (No *vs.* Yes)	0.91	0.51	1.63	0.76	0.99	0.57	1.73	0.98
Alcohol exposure (No *vs.* Yes)	1.21	0.76	1.94	0.40	1.09	0.70	1.71	0.68
Size (≤2 cm *vs.* >2 cm)	1.91	0.60	6.08	0.27	2.53	0.61	10.38	0.19
Stage (I *vs.* II)	2.42	1.10	5.32	0.02	2.02	0.93	4.41	0.07
Lymph nodes involved (No *vs.* Yes)	3.99	0.54	29.28	0.17	3.42	0.47	24.81	0.22
Grade (Low *vs.* High)	1.788	1.13	2.81	0.01	1.60	1.04	2.45	0.03
Margin status (Negative *vs.* Positive)	1.86	1.17	2.94	0.008	1.76	1.14	2.69	0.009
DLL3 (Low *vs.* High)	0.49	0.29	0.84	0.01	0.54	0.32	0.91	0.02
	Multivariate PFS (95%CI)				Multivariate OS (95%CI)			
Grade (Low *vs.* High)	1.59	0.977	2.58	0.06	1.63	1.03	2.58	0.03
Margin status (Negative *vs.* Positive)	1.99	1.24	3.19	0.004	1.93	1.24	3.00	0.004
DLL3 (Low *vs.* High)	0.46	0.25	0.82	0.009	0.58	0.34	1.01	0.05

PFS: progression-free survival; OS: overall survival; HR: hazard ratio; CI: confidence interval; *vs.*: *versus*; cm: centimeters.

**Table 2:** Clinicopathological characteristics of PDAC patients recruited in the study.

Clinical Characteristics	All Patients (N = 122) (%)
Age-yr	
≤65	33 (27)
>65	89 (73)
Median	72
Range	42
Sex	
Female	68 (55.7)
Male	54 (44.3)
Adjuvant treatment	
No	57 (46.7)
Yes	50 (41)
N/A	15 (12.3)
Grade	
Low	41 (33.6)
High	77 (63.1)
N/A	4 (3.3)
Vascular invasion	
No	59 (48.4)
Yes	53 (43.4)
N/A	10 (8.2)
Perineural invasion	
No	21 (17.2)
Yes	91 (74.6)
N/A	10 (8.2)
Margin status	
R0	65 (53.3)
R1	41 (33.6)
N/A	16 (13.1)
Pathologic T status	
T1	26 (21.3)
T2	43 (35.2)
T3	45 (36.9)
T4	1 (0.8)
N/A	7 (5.7)
Pathologic N Status	
N0	49 (40.2)
N1	52 (42.6)
N2	20 (16.4)
N/A	1 (0.8)
Stage TNM 8th edition	
IA	18 (14.8)
IB	13 (10.7)
IIA	15(12.3)
IIB	41 (41.8)
III	18 (14.8)
N/A	7 (5.7)
N/A: not available	

**Table 3:** Uni- and multivariate proportional hazard model for both progression-free and overall survival of patients from the validation cohort.

	Univariate PFS (95%CI)				Univariate OS (95%CI)			
	HR	Lower	Upper	*p*	HR	Lower	Upper	*p*
Age (<65 years *vs.* >65 years)	1.29	0.76	21.8	0.33	1.50	0.88	2.56	0.12
Sex (Male *vs.* Female)	1.41	0.88	2.27	0.15	1.35	0.85	2.14	0.20
Diabetes mellitus (No *vs.* Yes)	1.47	0.91	2.39	0.11	1.43	0.87	2.34	0.15
Symptoms at diagnosis (No *vs.* Yes)	1.75	0.91	3.34	0.08	2.11	1.04	4.28	0.03
Adjuvant treatment (Yes *vs.* No)	1.09	0.67	1.77	0.72	0.83	0.51	1.35	0.46
Size (≤2 cm *vs.* >2 cm)	2.27	1.21	4.26	0.01	1.84	1.04	3.26	0.03
Stage (I *vs.* II)	2.08	1.19	3.66	0.01	1.79	1.06	3.03	0.02
Lymph nodes involved (No *vs.* Yes)	1.81	1.10	2.97	0.01	1.20	0.75	1.90	0.44
Grade (Low *vs.* High)	1.22	0.74	2.01	0.42	1.31	0.81	2.14	0.26
Necrosis (No *vs.* Yes)	1.32	0.76	2.26	0.31	1.63	0.98	2.72	0.05
Vascular invasion (No *vs.* Yes)	1.10	0.69	1.78	0.66	0.88	0.55	1.41	0.59
Neural invasion (No *vs.* Yes)	0.95	0.51	1.74	0.87	0.92	0.52	1.62	0.78
Tumor DLL3 (Low *vs.* High)	0.55	0.34	0.87	0.01	0.59	0.37	0.93	0.02
	Multivariate PFS (95%CI)				Multivariate OS (95%CI)			
Symptoms at diagnosis (No *vs.* Yes)					2.12	1.00	4.50	0.05
Stage (I *vs.* II)	1.85	0.69	4.93	0.21	1.80	0.89	3.60	0.09
Size (≤2 cm *vs.* >2 cm)	1.64	0.77	3.49	0.19	1.66	0.83	3.34	0.15
Lymph nodes involved (No *vs.* Yes)	1.13	0.51	2.48	0.74				
Tumor DLL3 (High *vs.* Low)	0.41	0.23	0.71	0.02	0.44	0.255	0.76	0.004

**Table 4:** Association between tumor DLL3 protein expression and clinicopathologic parameters.

Clinical Characteristics	DLL3 Low	DLL3 High	*p*-Value
Age-years			0.616
≤65	18	15	
>65	43	44	
Sex			0.069
Female	39	28	
Male	22	31	
Adjuvant treatment			0.3
No	33	24	
Yes	18	31	
Grade			0.9
Low	21	20	
High	38	38	
Vascular invasion			0.39
No	31	27	
Yes	24	29	
Perineural invasion			0.007
No	16	5	
Yes	39	51	
Margin status			0.126
No affected (R0)	36	29	
Affected (R1)	16	24	
Pathologic T status			0.093
T1/2	29	39	
T3/4	27	19	
Pathologic N Status			0.029
N0	30	29	
N1/2	18	40	
Stroma DLL3			0.018
Low	37	23	
High	24	36	

## Data Availability

Data available upon request to correspondence authors.

## Supplementary Materials

The following supporting information can be downloaded at: https://www.mdpi.com/article/10.3390/biomedicines11102812/s1, Supplementary Figure S1: Survival analyses according to DLL3 protein expression in stroma tissues.
