# Supplementary material for: DLL3 Is a Prognostic and Potentially Predictive Biomarker for Immunotherapy Linked to PD/PD-L Axis and NOTCH1 in Pancreatic Cancer
Source: Biomedicines. 2023 Oct 17;11(10):2812. doi: 10.3390/biomedicines11102812 (PMC10604228; doi:10.3390/biomedicines11102812)
Supplement: Supplementary file 1 [file biomedicines-11-02812-s001.zip › biomedicines-2614647-supplementary.pdf]

biomedicines

MDPI

A

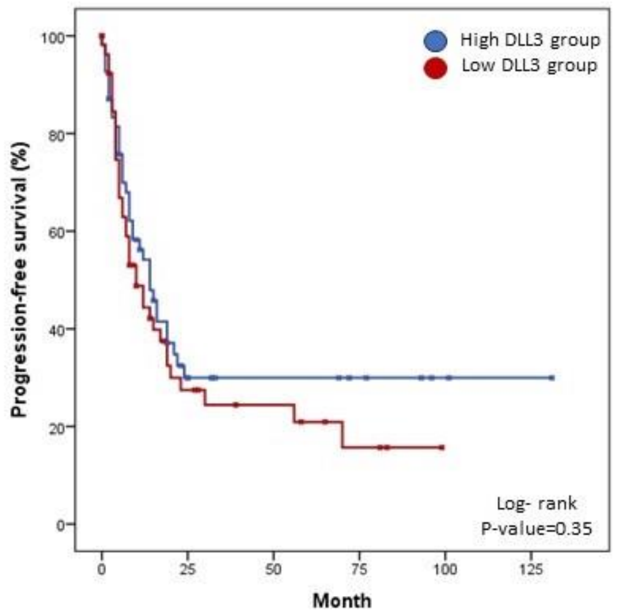

B

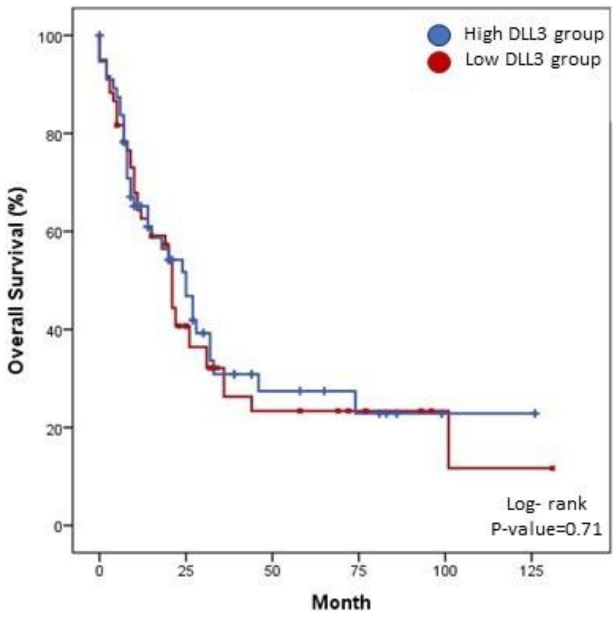

DLL3 Stroma (PFS)	Mean		Median	
	Estimate	CI 95%	Estimate	CI 95%
Low expression	45,9	(30,1- 61,8)	14,0	(9,3- 18,6)
High expression	28,2	(17,7- 38,7)	10,0	(5,2- 14,7)

DLL3 Stroma (OS)	Mean		Median	
	Estimate	CI 95%	Estimate	CI 95%
Low expression	39,5	(26,4- 52,7)	21,0	(18,9- 23,0)
High expression	44,0	(29,6- 58,5)	25,0	(16,2- 33,7)

Supplementary Figure S1. Survival analyses according to DLL3 protein expression in stroma tissues. (**A**) Progression-free survival analysis according to DLL3 protein expression. (**B**) Overall survival analysis according to DLL3 protein expression. The blue lines denote the high DLL3 expression arm. The red lines correspond to the low DLL3 expression patients.
